# Memories of near-death experiences: are they self-defining?

**DOI:** 10.1093/nc/niz002

**Published:** 2019-03-01

**Authors:** H Cassol, A D’Argembeau, V Charland-Verville, S Laureys, C Martial

**Affiliations:** 1 GIGA-Consciousness, GIGA Research Center, University of Liège, Liège, Belgium; 2 Coma Science Group, Department of Neurology, University Hospital of Liège, Liège, Belgium; 3 Department of Psychology, Psychology and Neuroscience of Cognition Research Unit, University of Liège, Liège, Belgium

**Keywords:** near-death experiences, near-death experiences-like, self-defining memories, autobiographical memory, Centrality of Event Scale

## Abstract

Some people report memories of near-death experiences (NDEs) after facing situations of impending death and these memories appear to have significant consequences on their lives (here referred to as “real NDE experiencers”; real NDErs). We assessed to what extent NDE memories are considered self-defining: memories that help people to define clearly how they see themselves. We screened 71 participants using the Greyson NDE scale (48 real NDErs and 23 NDErs-like who had lived a similar experience in absence of a threat to their life). Participants described their two main self-defining memories (SDMs). For each SDM, they completed the Centrality of Event Scale (CES) to assess how central the event is to their identity. The two subgroups did not differ regarding the proportion of NDErs who recalled their NDE (30 real NDErs out of 48 and 11 NDErs-like out of 23). Real NDErs and NDErs-like who recalled their NDE (*n* = 41) reported richer experiences as assessed by the Greyson NDE scale. Furthermore, these participants rated their NDE memory as more central to their identity as compared to other SDMs, and the richness of the NDE memory was positively associated to its centrality (CES scores). Overall, these findings suggest that the self-defining aspect of the experience might be related to its phenomenological content rather than its circumstances of occurrence. The self-defining status of NDE memories confirms that they constitute an important part of NDErs’ personal identity and highlights the importance for clinicians to facilitate their integration within the self.

## Introduction

Near-Death Experiences (NDEs) are profound psychological events with highly emotional and self-related content, generally occurring when people come close to death (Greyson 2000a). Typically, these experiences encompass affective, cognitive, transcendental, and paranormal elements such as, in order of frequency of occurrence: an intense feeling of peacefulness and pleasantness, an out-of-body experience, the vision of a very bright light, an altered time perception, the sensation of entering an unearthly world, or a sense of harmony and unity with the universe (Greyson 1983; Charland-Verville *et al.* 2014). Studies have highlighted that 6–23% of cardiac arrest survivors report a NDE, indicating that it is a frequent phenomenon (Parnia *et al.* 2001; Schwaninger *et al.* 2002). Intriguingly, similar experiences have been described after situations that do not imply any danger for physical or mental health (Charland-Verville *et al.* 2014). These experiences have been termed “NDEs-like” and have been described in contexts such as meditative states or syncope (Lempert *et al.* 1994; Beauregard *et al.* 2009). To date, no theory has been able to fully account for the whole NDE phenomenon. However, several hypotheses have been formulated regarding the role of psychological factors, notably higher dissociative symptoms and fantasy proneness in people who have lived these experiences (so-called NDE experiencers or NDErs; Greyson 2000b; Martial *et al.* 2018), as well as neuropsychological mechanisms (Jansen 1990; Timmermann *et al.* 2018).

Along with its particular circumstances of appearance and its mystical connotation, the NDE phenomenon seems to be characterized by a rich phenomenology and a sense of realness (Martial *et al.* 2017; Moore and Greyson 2017). Consequently, NDEs appear to have short- and long-term consequences on people’s lives (Greyson 1997; Van Lommel *et al.* 2001). Some authors have described a pattern of change comprising a reduced fear of death, a greater care and compassion towards others or a diminished value of material possessions (Ring 1984; Groth-Marnat and Summers 1998). Given their significance and consequentiality, NDE memories appear to share similarities with a particular type of autobiographical memories referred to as self-defining memories (SDMs; Blagov and Singer 2004). SDMs are emotionally intense, vivid, and frequently recalled memories that reflect important themes and conflicts in a person’s life (Singer and Salovey 1993). These emotional memories are the building blocks of identity and contribute, in particular, to the sense of self-continuity (Conway *et al.* 2004), which relies on the ability to consider oneself as an entity that extends back into the past and forward into the future (Chandler 1994). This ability is central to numerous processes such as planning future actions, giving meaning to new experiences or taking responsibility (Sani 2008).

SDMs can be characterized along four dimensions: (i) their specificity, which refers to the structure of the memory; (ii) the presence of meaning making, which reflects the individual’s propensity to derive personal meaning and reflect upon the experience; (iii) their content, which makes reference to the primary concern of the experience; and (iv) the associated affect, which represents the emotions felt upon recall (see Table 1 for a more detailed description; Blagov and Singer 2004). From a clinical perspective, scholars have shown that the recall of emotionally positive SDMs as well as the ability to integrate a lesson or derive meaning from an experience are related to higher levels of psychological adjustments in healthy individuals (Blagov and Singer 2004; Wood and Conway 2006). Hence, careful consideration should be given to the development of these capacities of integration and autobiographical reasoning, especially when attempting to (re)build a sense of self-continuity (Inder *et al.* 2008). This integration process seems particularly important after intense life-threatening experiences such as NDEs.

**Table 1:** The four dimensions of SDMs (Blagov and Singer 2004; McLean and Fournier 2008)

Dimension	Description		
Specificity	Specific	Non-specific	
	*Specific memories* are characterized by uniqueness of occurrence and limited duration in time (<24 h).	*Extended memories* include events that occurred over an extended period of time (>24 h).	*Generic memories* comprise events that occurred repeatedly over time.
	Example: “The day I broke my leg during gym class in second grade.”	Example: *“*My skiing holidays in Switzerland when I was 10 years old.”	Example: “The huge annual Christmas meals at grandma’s.”
Autobiographical reasoning	Autobiographical reasoning is notably studied as a dimension of SDMs and consists in reflecting on the implications and the personal meaning of the event (Singer and Bluck 2001). This meaning making may relate to lesson learning and/or insight gaining (Thorne and McLean 2001).		
Event content	SDMs have been classified into seven distinct categories (Thorne and McLean 2001): Life-threatening This category includes events with issues of life and death. The mentioned emotions are generally fear or sadness. These events may correspond to: Death or serious illness/injury of someone else Serious accidents or illnesses to oneself Physical assaults Rapes, attempted rapes, or sexual abuses to oneself Not classifiable Recreation/exploration Narratives corresponding to this category center on recreational activities such as hobbies or travels. Relationship Relationship events comprise experiences in which interpersonal relationships are emphasized, such as a first love or a separation. Achievement/mastery This category groups events relating to arduous attempts at accomplishment, for example winning a competition, passing or failing an exam. Guilt/shame This subset of events includes memories focusing on notions of “doing right” vs. “doing wrong” (e.g. feeling guilty about lying or hurting someone). Drug/alcohol abuse Narratives classified in this category are centered on drugs, tobacco and alcohol, for recreational use as well as suicide attempts. Not classifiable Not classifiable events refer to experiences that do not fit into any of the previous categories.		
Affect	This dimension corresponds to the affective response triggered by the retrieval of the memory (Blagov and Singer 2004).		

This study therefore seeks to determine if NDE memories may be considered as self-defining. First, because of their documented life-transforming effects and their reported importance (Greyson 1997), we predicted that a majority of NDErs would describe their NDE as one of their two main SDMs. To do so, NDErs completed an online survey on their SDMs. In order to determine whether the potential self-defining dimension of NDEs is due to their phenomenal content or their circumstances of appearance (i.e. presence or absence of impending death), we also included a subgroup of NDErs-like (i.e. people who experienced a NDE in absence of life threat). Second, we expected that NDEs memories would be considered as more central to NDErs’ identity and life story as compared to the other recalled SDM. To test this hypothesis, the Centrality of Event Scale (CES; Berntsen and Rubin 2006), which assesses how central an event is to a person’s identity, was administered. We also looked at the association between the intensity of the NDE, as measured by the Greyson NDE scale developed to quantify the self-reported richness of the experience (Greyson 1983), and its centrality to NDErs’ selves. Finally, we explored the four dimensions of reported SDMs with a particular attention to meaning making and affect, which are crucial to psychological well-being.

## Materials and Methods

### Participants

Participants were initially recruited following calls for NDE testimonies via the websites, the appearances in local media, and the publications of the International Associations for Near-Death Studies (IANDS) and the Coma Science Group (GIGA-Consciousness, University and University Hospital of Liège, Belgium). People who contacted us to share a written narrative of their experience were mailed questionnaires including the Greyson NDE scale (Greyson 1983), used to identify the presence of a NDE memory by assessing the presence of specific affective, cognitive, transcendental, and paranormal features. This 16-item multiple-choice validated scale enables to quantify the richness of the experience (i.e. total score ranging from 0 to 32) and allows a standardized identification of NDEs (i.e. cut-off score of 7). For each item, scores are arranged on an ordinal scale ranging from 0 to 2 (i.e. 0 = “not present,” 1 = “mildly or ambiguously present,” and 2 = “definitively present”; Greyson 1983). Additionally, they completed questionnaires requesting socio-demographic information (gender, age at interview, and religious belief at the time of the NDE—religious or non-religious), their age when they experienced the NDE, the time elapsed since the NDE, and the circumstances of appearance of their NDE. On this basis, they were assigned to two subgroups: a “real NDE” subgroup (i.e. experiences equal or above the cut-off score of 7 that occurred following a severe brain injury accompanied by a period of coma >1 h and an hospitalization in an intensive care unit) or a “NDE-like” subgroup (i.e. similar phenomenological experiences equal or above the cut-off score of 7 that occurred in situations where there was no genuine threat to the individual’s life; Charland-Verville *et al.* 2014). Once the participants were divided into two subgroups, they were re-contacted and invited to participate in another study focusing on SDMs. The final study sample was composed of 48 individuals who reported having lived a “real NDE” and 23 individuals who described a “NDE-like.”

The study was approved and carried out in accordance with the recommendations of the ethics committee of the Faculty of Medicine of the University of Liège. All participants completed a written informed consent in accordance with the Declaration of Helsinki.

### Materials

#### Self-defining memory task

Participants were invited to describe their two main SDMs. It was not specified whether or not one of them should be their NDE since we wanted to determine the proportion of NDErs who would spontaneously mention their NDE as one of their main SDMs. All participants wrote down their two main SDMs in any order (i.e. without constraints regarding the chronology or the subjective importance of the recalled memories) and with no limit regarding the number of words. To do so, they received a written definition of a SDM: (i) the memory has to be at least 1-year-old (in order to better gauge the impact of an event, one has to have a sufficient hindsight regarding what he/she has experienced; it should be underlined that this criterion could not have impacted the number of NDEs recalled since all NDErs had experienced their NDE more than one year before completing the survey); (ii) it has to be a very clear memory of an important event that was personally experienced; (iii) it helps understanding who one is as an individual; (iv) it relates to a personally significant and enduring theme or concern, and it is linked to other memories that share the same theme; (v) it generates strong feelings, no matter the valence; and (vi) it has been recalled a great number of times (adapted from Singer and Moffitt 1991–1992; Blagov and Singer 2004).

#### Affect

Participants indicated on a 7-point Likert scale ranging from −3 (very negative) to +3 (very positive): (i) emotions felt during the event and (ii) emotions felt while remembering the event and thinking about its consequences on their lives (questions derived from the validated Memory Characteristic Questionnaire; Johnson *et al.* 1988).

#### Centrality of event scale

This instrument measures how central an event is to a person’s identity and life story, with 20 items rated on a 5-point scale (from 1 = totally disagree to 5 = totally agree). Items 1, 2, 4, 9, 12, 13, 17, and 20 have been developed to assess whether an event has become a reference point for the generation of expectations and attribution of meaning to other events in one’s life story. Items 3, 5, 6, 7, 8, and 19 were designed to assess whether an event is considered as a central component of the person’s identity. Finally, items 10, 14, 15, 16, and 18 address whether the event is considered by the respondent as a turning point in his/her life story (Berntsen and Rubin 2006).

### Procedure

After initial recruitment (see Participants section), participants of both subgroups (“real NDE” and “NDE-like”) were invited to participate in a study on SDMs. They received an email with a link to an online survey: (i) they completed an online consent form including a description of the study; (ii) they answered items related to socio-demographic data; (iii) they completed the SDM task; (iv) finally, for each of their two SDMs, they rated the associated affect and completed the CES. The order of rating of the two SDMs was randomized: part of the participants rated the first recalled SDM as a first step and then the second recalled SDM, while the other participants rated their SDMs in the reverse order. The order of SDM rating was randomized to circumvent a possible bias: we assumed that most participants would recall their NDE memory first and we wanted to minimize possible order effects in describing and assessing SDMs.

### Analyses

Results were considered to be significant at an alpha of 0.05 and were expressed as mean (*M*) ± standard deviation (SD) for normally distributed quantitative variables and as median (*Mdn*) [inter-quartile range (IQR)] for quantitative variables with a skewed distribution. Categorical variables were expressed as counts and proportions (%). Data analyses were carried out using R statistical software (R 3.4.1).

#### Demographics and questionnaires

As a first step, we compared the gender and religious beliefs ratios between “real NDEs” and “NDEs-like” by means of a Fisher’s exact test with contingency tables. Student’s *t*-tests were used to compare reported intensity of the NDE (Greyson NDE scale total scores), age at NDE, and age at interview between subgroups. Distributions being skewed, differences for time since NDE were analyzed using Wilcoxon–Mann–Whitney *U* tests. Finally, percentages of NDErs who recalled their NDE were calculated for each subgroup and a Fisher’s exact test was performed to compare ratios between subgroups.

**Figure 1.**
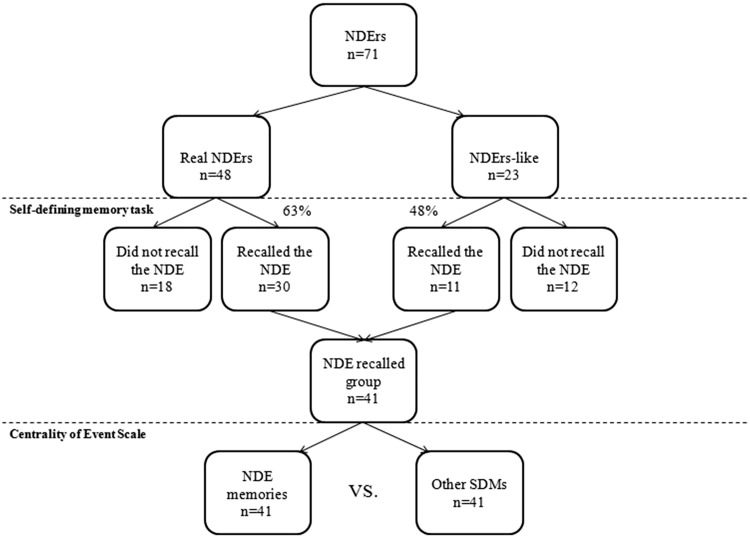
Flowchart representing the distribution of participants within the different subgroups.

As a second step, all participants were divided into two subgroups depending on whether or not they recalled their NDE (no matter its context of occurrence). We compared the gender ratio between the “NDE recalled” and the “NDE not recalled” subgroups with a Fisher’s exact test. Intensity of the NDE (Greyson NDE total scores), age at NDE, age at interview and time since NDE were compared by means of Student’s *t*-tests. Figure 1 represents the flowchart of the distribution of NDErs within the different subgroups.

#### Dimensions of self-defining memories

First, we examined the centrality of events for identity and life story (as assessed by the CES scale) in the “NDE recalled” subgroup. Differences in CES total scores between the NDE memory and the other SDM were assessed using a Student’s *t*-test. Next, we examined associative strength between CES and Greyson NDE scale total scores for the NDE memories using a Spearman’s correlation.

Second, ratings of **affect** were compared between NDE memories and other SDMs, using a Wilcoxon–Mann–Whitney *U* test.

Third, two coders (HC and CM) scored the four dimensions of all reported SDMs (*n* = 142). The **specificity** of each SDM was classified in three categories: (i) specific events that happened in a particular time and place and lasted less than 24 h; (ii) extended events that lasted longer than a day; and (iii) generic events that occurred repeatedly. The number of observed agreements was 138 out of 142 (97.18%), which corresponds to a Cohen’s kappa coefficient of 0.89 (95% confidence intervals 0.79–1). The presence (score of 1) or absence (score of 0) of **meaning making** in each narrative was also determined. The number of observed agreements was 135 out of 142 reported SDM narratives (95%), which corresponds to a Cohen’s kappa coefficient of 0.90 (95% confidence intervals 0.83–0.97). To examine whether NDE memories differed from the other SDMs in terms of meaning making, a Pearson’s chi-squared test was used to assess frequency distribution of meaning making among the two types of memories. Finally, coders proceeded to the rating of the **content** of SDMs based on the classification proposed by Thorne and McLean (2001). This classification includes seven major categories: (i) life-threatening events, (ii) recreation/exploration, (iii) relationship, (iv) achievement/mastery (either with positive or negative outcomes), (v) guilt/shame, (vi) drug/alcohol abuse, and (vii) “not classifiable.” Additionally, a subclassification of life-threatening events was used, as suggested by Thorne and McLean (2001): (a) death or serious illness/injury of someone else (person or animal), (b) serious accidents or illnesses of oneself, (c) physical assault, (d) rape, attempted rape, or sexual abuse (to oneself), (e) “not classifiable” life-threatening events. The number of observed agreements for all SDMs was 135 out of 142 (95% of the observations), which corresponds to a Cohen’s kappa coefficient of 0.94 (95% confidence intervals 0.90–0.98). For all dimensions, discrepancies between raters were discussed in order to reach a final classification of all the narratives.

## Results

### Demographics and questionnaires

The “real NDE” subgroup (*n* = 48) included NDEs that occurred following a life-threatening situation such as anoxia (e.g. cardiac arrest, near-drowning; *n* = 19), trauma (e.g. motor vehicle accident, falls; *n* = 14), and other events (non-traumatic events such as complication during surgery; *n* = 15). The “NDE-like” subgroup (*n* = 23) included NDEs that occurred following a non-life-threatening event such as sleep (*n* = 5), syncope (*n* = 5), drug and alcohol consumption, (*n* = 3) or other non-life-threatening situations (e.g. grief, migraine; *n* = 10). No significant differences were found between “real NDEs” and “NDEs-like” in terms of gender, Greyson NDE total scores, age at NDE, age at interview, and time since NDE (see Table 2).

**Table 2:** NDErs’ Greyson total scores and demographic characteristics (*n* = 71)

	Real NDE (*n* = 48)	NDE-like (*n* = 23)	*P*	Effect size	NDE recalled (*n* = 41)	NDE not recalled (*n* = 30)	*P*	Effect size
Gender—female (%)	32 (67)	18 (78)	0.317	0.119^b^	28 (68)	22 (73)	0.646	0.055^b^
Religious (%)	35 (73)	14 (61)	0.411	−0.122^b^	**33 (80)**	**16 (53)**	**0.019**	**−0.29**^b^
Greyson total score	17 (5)	15 (5)	0.124	**−**0.4	**17 (5)**	**14 (5)**	**0.008**	**−0.6**
Age at NDE	28 (16)	34 (17)	0.207	0.368	28 (19)	33 (19)	0.199	0.263
Age at interview	57 (14)	59 (11)	0.558	0.152	57 (14)	58 (12)	0.635	0.076
Time since NDE in years	31 (12–41)^a^	26 (10–38)^a^	0.376	−0.106^c^	29 (16)	25 (17)	0.365	−0.243

Quantitative variables are summarized using the *M*(SD), except for data with ^a^representing *Mdn*(IQR). The effect size is expressed as G_Hedges_, except for data with ^b^which are expressed as *ϕ* and data with ^c^which is expressed as *r*.

Results in bold are significant.

Overall, 58% of the total sample recalled the NDE as one of their two main SDMs (41 participants out of 71), respectively 30 NDErs out of 48 in the “real NDE” subgroup vs. 11 NDErs out of 23 in the “NDE-like” subgroup. The Fisher’s exact test did not show a significant difference between subgroups regarding the ratio of participants recalling the experience (*P* = 0.307; *ϕ =* −0.139).

“NDE recalled” and “NDE not recalled” subgroups did not significantly differ in regard to gender, age at NDE, age at interview and time since NDE. In contrast the “NDE recalled” subgroup showed higher total scores on the Greyson NDE scale (see Table 2).

### Dimensions of SDMs

**Figure 2.**
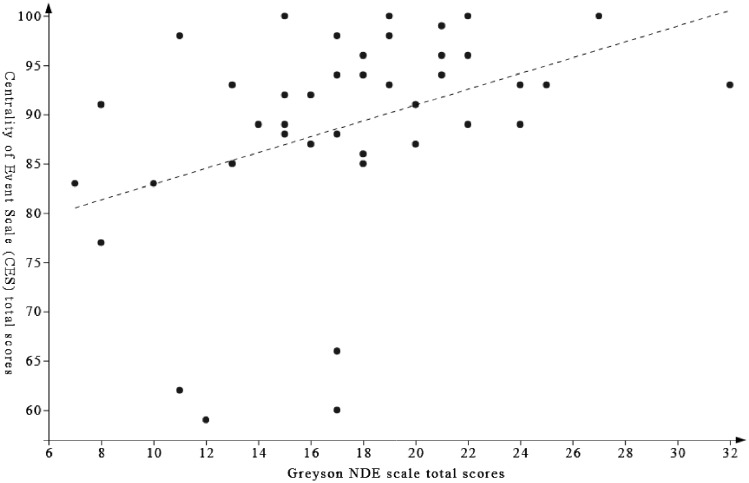
Association between CES total scores for the NDE memory and Greyson NDE scale total scores within the NDE recalled subgroup (r_*s*_ = 0.48, *P* = 0.001).

Analysis of the CES scores carried out on the “NDE recalled” subgroup (*n* = 41; 30 real NDErs and 11 NDErs-like) showed that NDE memories received higher CES total scores (*Mdn *=* *92, IQR = 87–96) than other SDMs (*Mdn *=* *75, IQR = 59–84) (*P* < 0.001; *r* = −0.574). Moreover, a significant positive correlation was found between CES scores for the NDE memory and Greyson NDE scale total scores (r_*s*_ = 0.48, *P* = 0.001; Fig. 2).

Regarding **affect**, the analyses showed that emotions felt during the event were more positive for NDE memories (*Mdn *=* *7, IQR = 6–7) compared to other SDMs (*Mdn *=* *4, IQR = 1–6) (*P* < 0.001; *r* = −0.430). Moreover, emotions felt upon recall were also considered more positive for NDE memories (*Mdn *=* *7, IQR = 6–7) compared to other SDMs (*Mdn *=* *6, IQR = 4–7) (*P* < 0.001; *r* = −0.346).

The classification of the **specificity** of all SDMs (*n* = 142) indicated that participants reported 122 specific SDMs (<24 h; 86%), 14 extended SDMs (>24 h; 10%), and 6 generic SDMs (repeated events; 4%). All NDE memories were specific.

**Meaning making** was detected in 62 of the 142 SDMs (44%). The difference in ratio of meaning making between NDE memories (21 out of 41; 51%) and other SDMs (41 out of 101; 41%) was not statistically significant (*P* = 0.267; *ϕ *= 0.097).

**Figure 3.**
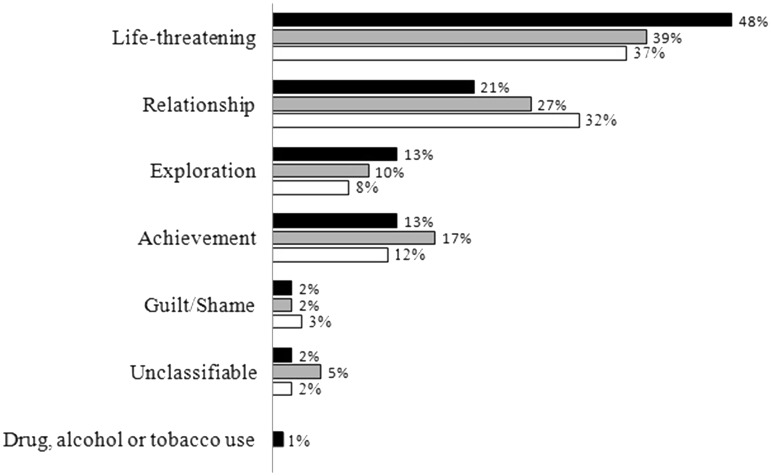
Classification of SDMs within the major event categories proposed by Thorne and McLean (2001). In black: distribution of all reported SDMs (*n* = 142). In gray: distribution of the other (non-NDE) SDM in the “NDE recalled” subgroup (*n* = 41). In white: distribution of the SDMs in the “NDE not recalled” subgroup (*n* = 60).

Finally, regarding memory **content**, NDEs recalled by real NDErs (*n* = 30) were all classified as serious accidents or illnesses to oneself (despite containing additional transcendental and paranormal components as compared to other life-threatening events, as assessed by the Greyson NDE scale) and NDEs recalled by NDErs-like were classified as exploration events (i.e. experiencing an unexpected spiritual moment; *n* = 10) or events relating to drug use (*n* = 1). The classification of all reported SDMs (including NDEs and NDEs-like; *n* = 142) consisted of 68 memories of life-threatening events (48%), 30 memories relating to relationships (21%; e.g. life-changing encounter, child birth, or family/friend reunion after a long separation), 19 memories centered on exploration (13%; e.g. facing personal challenges), 18 memories that emphasize achievement (13%; e.g. professional recognition and promotion or difficult child birth), and 3 memories about feelings of guilt/shame (2%), 3 unclassifiable events (2%), and 1 event relating to drug use (1%). Subclassification of the 68 life-threatening events included 42 events of serious accidents or illnesses to oneself (62%; e.g. learning to live in an handicapped body after severe accident or survival during times of war), 18 events of deaths or serious illnesses/injuries of someone else (26%; e.g. death in the family or illness of a child), 7 events relating to physical assault (10%; e.g. domestic violence), and 1 unclassifiable event (2%). The distribution of the 142 memories within the seven main categories is presented in Fig. 3.

Overall, when examining the number of participants who recalled each type of content, we found that NDE memories constituted by far the most important category, as they were recalled by 41 participants (58% of the participants). The second most frequently reported subcategory of event was the “death of a family member” which was reported by 18% of participants (*n* = 13), followed by a “life-changing encounter” (*n* = 7; 10%), “domestic violence” (*n* = 6; 8%), “family/friend reunion after a long separation” (*n* = 5; 7%), and “learning to live in an handicapped body after severe accident”, and “confrontational relationships” (both reported by four participants; 6%). “Academic success,” “child births,” “facing personal challenges,” and “forced exile during times of war” were each reported by three participants (4%). “Difficult child birth,” “getting out of a depression,” “illness of a child,” “moment of extreme shame,” “physical assault” (as an aggressor), and “professional promotions” were each reported by two participants (3%).

The distribution of the other (non-NDE) SDMs (*n* = 41) reported by the “NDE recalled” subgroup (i.e. real NDErs and NDErs-like who recalled their NDE as one of their two main SDMs) among the 7 major categories included 16 life-threatening events (39%), 11 events relating to relationships (27%), 7 events that emphasize achievement (17%), 4 events centered on exploration (10%), 2 unclassifiable events (5%), and 1 event relating to guilt/shame (2%). The drug, alcohol, and tobacco use category was not represented in this subgroup. Subclassification of the 16 life-threatening events included 7 events of serious accidents or illnesses to oneself (44%), 6 events of deaths or serious illnesses/injuries of someone else (37%), and 3 events relating to physical assault (19%). The other subcategories were not represented in the subgroup (see Fig. 3).

Lastly, the distribution of the SDMs (*n* = 60) reported by the “NDE not recalled” subgroup (i.e. real NDErs and NDErs-like who did not recall their NDE as one of their two main SDMs). This classification comprised 22 life-threatening events (37%), 19 events relating to relationships (32%), 7 events that emphasize achievement (12%), 5 events centered on exploration (8%), 2 events relating to guilt/shame (3%), and 1 unclassifiable event (2%). The drug, alcohol, and tobacco use category was not represented in this subgroup. Subclassification of the 22 life-threatening events included 12 events of deaths or serious illnesses/injuries of someone else (55%), 5 events of serious accidents or illnesses to oneself (23%), 4 events relating to physical assault (18%), and 1 unclassifiable event (4%). The other subcategories were not represented in the subgroup (see Fig. 3).

## Discussion

This study investigated whether the memory of a NDE is considered as self-defining. Furthermore, to disentangle between the roles of the nature of the experience and its circumstances of appearance, real NDErs (i.e. presence of a life-threatening event) and NDErs-like (i.e. absence of life threat) described their two most significant SDMs. In line with our hypothesis, 58% of the entire sample recalled their NDE as a SDM, respectively 63% of real NDErs and 48% of NDErs-like. Analyses did not show significant differences in proportions between subgroups. Moreover, when looking closely at the content of NDEs, results indicated that NDErs who selected their NDE as a SDM had lived richer experiences, as measured by the Greyson NDE scale (Greyson 1983). Indeed, it is likely that NDEs with more cognitive, affective, transcendental, and/or paranormal characteristics are experienced more intensely, leading to memories that are more likely to be considered self-defining. Additionally, we found a larger proportion of religious NDErs among participants who recalled their NDE. A possible explanation for this latter finding is that the NDE experience might be perceived as more meaningful for religious NDErs, thus being more likely to be part of their identity. Overall, our results suggest that the self-defining aspect of the NDE memory could be related to its particular phenomenological content rather than its circumstances of occurrence, although future studies are required to clarify/confirm this latter point. Altogether, these results support the view that NDE memories constitute an important part of NDErs’ personal identity and are self-defining. This proposition is also consistent with previous results showing that two-thirds of those who experienced a NDE during a close brush with death describe a substantial impact of this experience on their lives (Noyes 1980). Yet, it has to be borne in mind that the fact that some NDErs did not recall their NDE does not necessarily mean that this experience is not considered self-defining. Indeed, most people can generally recall more than two SDMs and it might be that a higher proportion of NDErs would recall their NDE if they were asked to recount more than two SDMs.

When considering participants who reported their NDE, we found a significant difference between the NDE memory and their other SDM regarding the CES total scores, the memory of the NDE being rated as more central to NDErs’ identities. Our results therefore suggest that NDE memories can be regarded as cornerstones in NDErs’ lives and that they might color the way they understand other experiences or the hardship in their lives. Additionally, richer (in terms of the number of reported features) NDEs were considered as more central to NDErs’ selves. Although this correlation does not demonstrate causality, it is reasonable to hypothesize that richer NDEs may lead to a memory that is more central to the person’s identity. Considering that the richness of the experience appears to be associated with NDErs’ traits (e.g. fantasy proneness; Martial *et al.* 2018), future studies should investigate to what extent NDE richness might also influence their interpretation and integration into the life story.

When taking a closer look at the dimensions of memories, we found that SDMs were globally pleasant and that NDE memories, in particular, were associated with positive affect upon recall. These results are consistent with the view that NDEs are typically associated with highly positive emotions (Greyson 1983). Regarding the specificity of reported SDMs, we observed that a large majority of memories (86%) involved specific events rather than extended or generic events. This high percentage can be partly explained by the fact that life-threatening events are typically specific. Lardi *et al.* (2010) have indeed highlighted that life-threatening events were the most specific memories, but they also noted that these events were typically associated with low positive affect and strong negative affect. Therefore, real NDEs seem to be a particular subtype of specific life-threatening events that has the particularity to be associated with positive affects upon recall.

Concerning memory content, half of the sampled memories referred to life-threatening events. The second most frequent type of memories were those in which interpersonal relationships are emphasized, followed by memories of events relating to exploration, memories highlighting effortful endeavor at mastery or accomplishment, and finally events encompassing guilt/shame and drug use themes. With the exception of life-threatening and exploration events that are unsurprisingly frequent in our sample given NDEs and NDEs-like events, the distribution of SDMs into the different categories is similar to some other studies in which memories relating to relationships and achievement concerns were largely represented (e.g. Lardi *et al.* 2010). In this context, it should also be emphasized that real NDEs memories appear as a unique and specific subtype of “serious accidents or illnesses to oneself” as they comprise distinguishable affective, cognitive, transcendental, and paranormal features, as compared to other events belonging to this subcategory.

In regard to meaning making, it is interesting to note that a majority of SDM narratives did not include meaning making (i.e. 44% of all reported SDMs included meaning making). Additionally, the difference in the prevalence of meaning making between NDE memories (51%) and other SDMs (41%) did not reach significance, which might suggest that autobiographical reasoning could partly depend on the individual rather than on the type of memory (Rubin *et al.* 2018). The relatively low proportion of meaning making for NDEs is somewhat surprising given that it is considered crucial for psychological adjustment; indeed, the ability to reflect upon one’s life experiences has been associated with higher levels of socio-emotional maturity and is developed in psychotherapy to improve introspection abilities and build a unified self (Inder *et al.* 2008). Therefore, the development of psychological interventions aiming at facilitating the integration of NDEs into the sense of self and identity might be fruitful for some individuals who have experienced this impactful event.

Finally, some limitations of this study should be acknowledged. Given the relative scarcity of NDEs, we were limited in the recruitment of our participants and our sample is relatively small. Larger studies are therefore required to confirm these results. Besides, because NDErs voluntarily contacted us, our sample suffers from a self-selection bias and one must bear in mind that, considering the mystical connotation of such experiences and the fact that they may be perceived as distressing, some people might feel uncomfortable to share these events. Another possible bias may have arisen from the fact that some participants might have guessed the objectives of the study and recalled their NDE accordingly or, on the contrary, might have felt that we wanted them to provide two memories other than their NDE. Nevertheless, after debriefing, no indication of this potential bias was observed. Finally, to exclude a possible influence of the context of events, future studies should also include a subgroup of individuals who have lived a close brush with death without experiencing a subjective NDE.

## Conclusion

The self-defining status of NDE memories reinforces the importance for caregivers to detect the presence of such an event in order to ensure, or at least facilitate, its later integration into NDErs’ selves. As a matter of fact, SDMs play a key role in the construction and maintenance of a coherent sense of self-continuity, which appears to be positively associated to psychological adjustment (Chandler *et al.* 2003). Highly accessible and vivid personal memories contribute to the construction of life narratives and, in this way, enable the stabilization of the sense of self and identity (Baerger and McAdams 1999). However, the outcome of this process is not systematically positive (Berntsen *et al.* 2003). A highly negative, unforeseen and relatively uncommon event might have an unfavorable influence on the interpretation of other experiences as well as on expectations about future events, and therefore can be harmful to mental health (Berntsen *et al.* 2003). Thereupon, it is reasonable to expect that atypical experiences of close brushes with death, such as NDEs, become reference points that could potentially lead to deleterious outcomes such as lower senses of self-continuity typically associated with anxiety and negative affect (Chandler *et al.* 2003). Overall, our study adds to the growing literature demonstrating the significant consequences of NDEs and highlights the fact that they deserve careful consideration.

## Data availability

Data are available upon request.

*Conflict of interest statement*. None declared.
